# Supplementary material for: Transient hydrodynamic effects influence organic carbon signatures in marine sediments
Source: Nat Commun. 2018 Nov 8;9:4690. doi: 10.1038/s41467-018-06973-w (PMC6224525; doi:10.1038/s41467-018-06973-w)
Supplement: Supplementary file 1 — Supplementary Information [file 41467_2018_6973_MOESM1_ESM.pdf]

Supplementary Information

Transient hydrodynamic effects influence organic carbon signatures in marine sediments (Magill et al.)

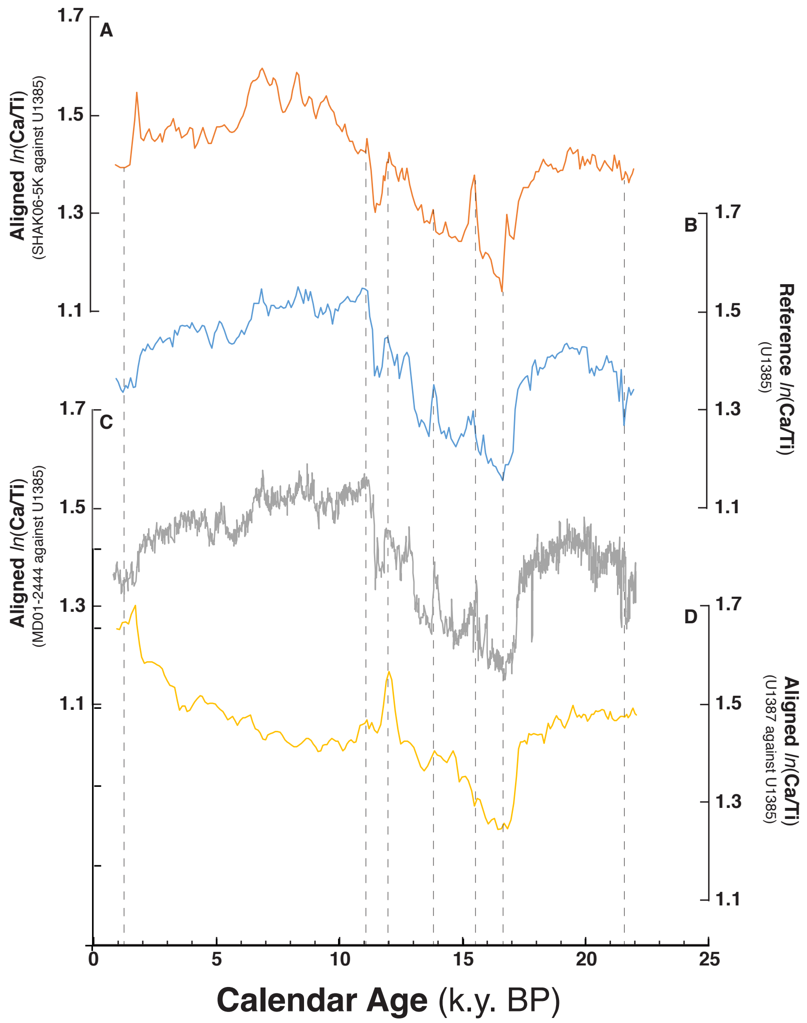

Supplementary Figure 1: Stratigraphic alignment of SHAK06-5K and key piston cores against U1385. Tie-points are indicated by grey (vertical) dashed lines. Bulk sediment XRF data at SHAK06-5K (Supplementary Data 2), MD01-2444 (ref. 1), U1387 (ref. 2) together were stratigraphically aligned to calcium-to-titanium ratios (Ca/Ti) for at U1385 (37.571 °N, 10.126 °W, 2578 mbsl)[1]. Previous studies establish down-core alignment between U1387 and U1386 and U1389 (refs. 2,3). Associated down-core records of these ratios were log-transformed because log-ratios of element intensities are simple linear algorithms of logarithmic concentration values[4], and facilitate comparisons of data derived from different instruments[1]. Previous studies suggest down-core sediment Ca/Ti ratios at Iberian margin sites reflect the relative influx of biogenic (Ca) as compared to detrital (Ti) materials, and both share a strong positive correlation with regional foraminiferal (planktonic) $\delta^{18}O$ temperature reconstructions and Greenland ice-core $\delta^{18}O$ trends[5].

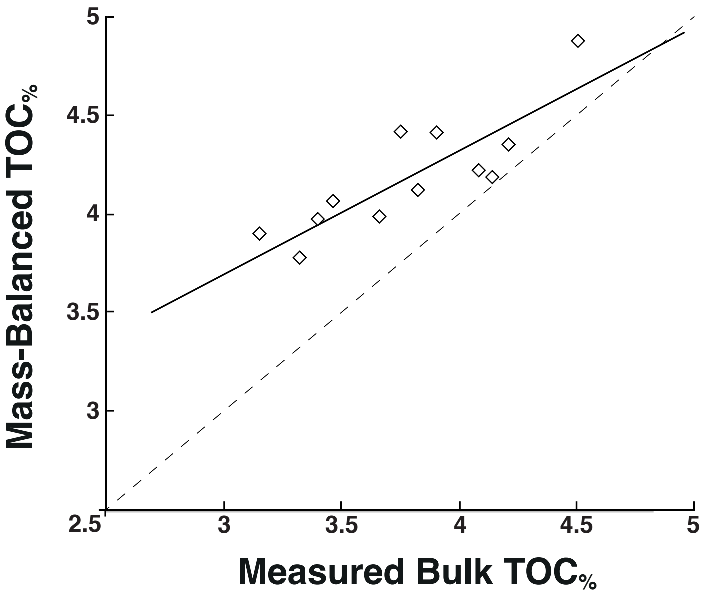

Supplementary Figure 2: Cross-plot of the 'mass-balanced' percent total organic carbon ($TOC_{\%}$) against the measured bulk sediment $TOC_{\%}$ (c.f., Supplementary Data 1). Mass-balance was achieved by pairing grain-size specific contributions to fractional bulk lithogenic matter (L_x, where x represents discrete sediment fractions), and the measured fractional bulk organic carbon content (B_x, where x represents a discrete sediment fraction):

$$L_C B_C + L_{FS} B_{FS} + L_{CS} B_{CS} + L_S B_S$$

Sediment fraction abbreviations: clay [C], fine silt [FS], coarse silt [CS], and sand [S].

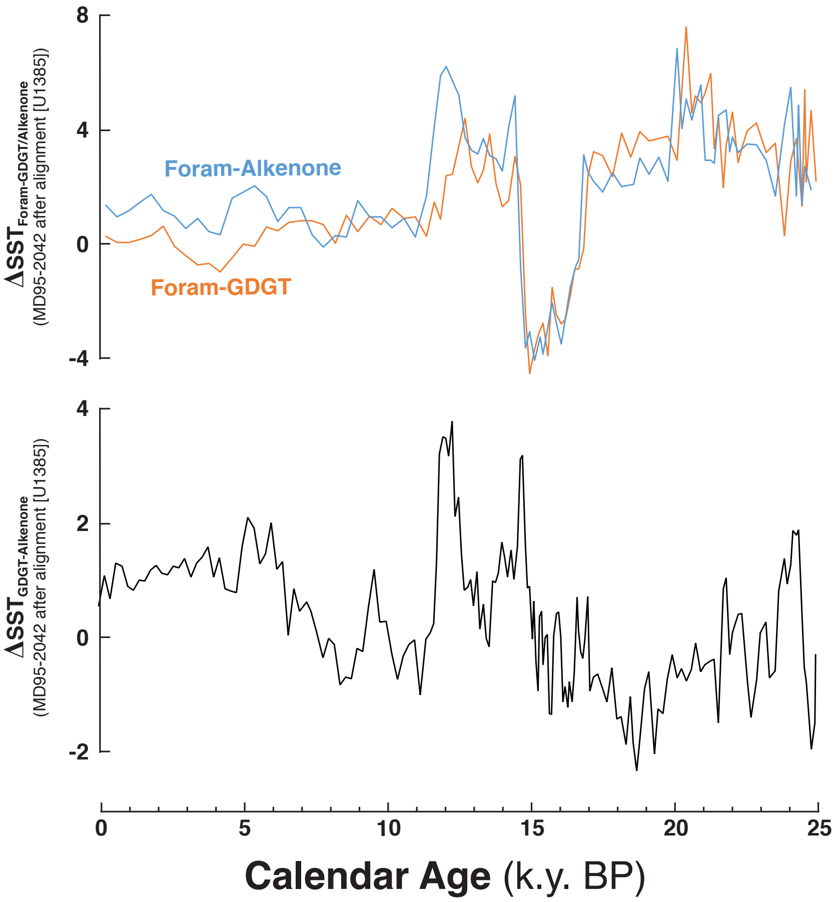

Supplementary Figure 3: Comparative differences in down-core records derived from at MD95-2042 for sea-surface temperature reconstructions vis-à-vis alkenones[6] (U^{k}_{37}), GDGTs[6] (TEX_{86}), and foraminiferal (*Globigerina bulloides*) tests[7]. Reconstructed SSTs are derived from identical down-core sample depths at MD95-2042, thus removing potential difficulties with between-core alignment biases (i.e., apparent lead/lag phase relationships are independent from absolute age uncertainties)[4].

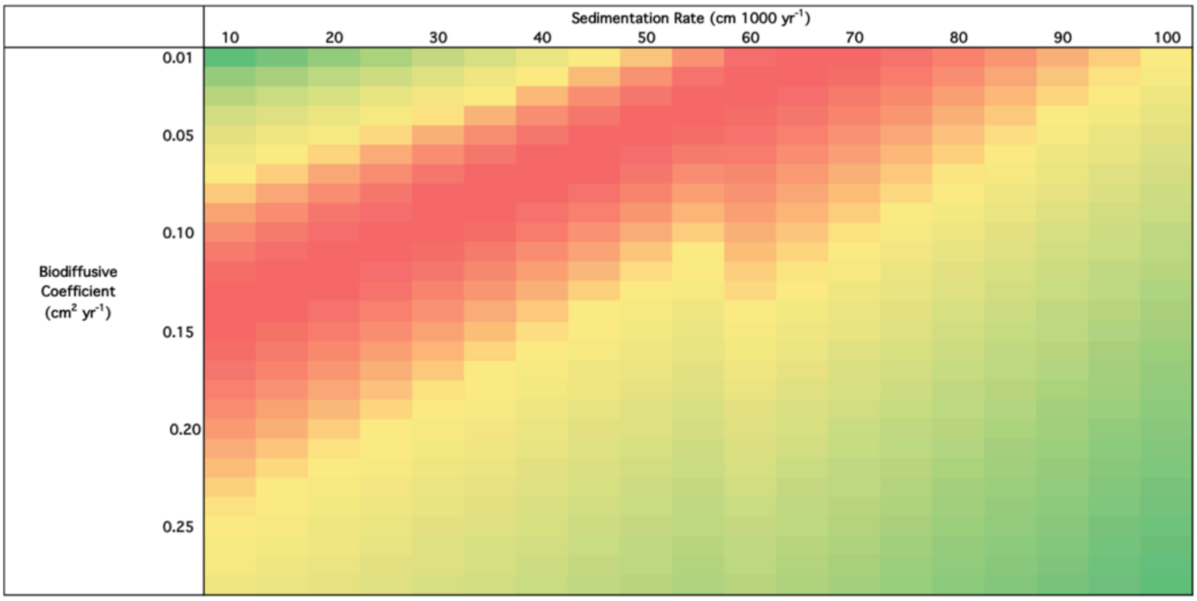

Supplementary Figure 4: Heat map optimizing parameter solutions for modelled biodiffusion. In this heat map, warmer colors (red) indicate the optimal solution pairs for sedimentation rate (S; cm kyr^{-1}) and the biodiffusive coefficient (D_b; cm^2 yr^{-1}) given ^{210}Pb activity at depth zero (A_0 = 44.5 dpm g^{-1}). The parameters were optimized by minimizing the sum of the negative log-likelihood between observed and modeled ^{210}Pb activity (A_x) for each depth (x) using the formula[8]: $A_x = A_0 e^{x(S-\sqrt{S^2+4\lambda D_b})(2D_b)^{-1}}$

Supplementary Table 1: Radiocarbon data of foraminifera and total organic carbon (TOC) for co-deposited bulk sediments and for respective clay (<2 um), fine silt (2-10 um), coarse silt (10-63 um), and sand (>63 um) fractions in sediments sampled from at SHAK06-5K. Analytical uncertainties are reported as 1-sigma standard deviation (s.d.). Dynamic reservoir ages were calculated from Skinner *et al.* (2014) based on correlative calendar year age estimates using CALIB 6.0. *n.d.* = not determined.

SHAK06-5K	*Globigerina bulloides*							
**Core Depth** (cm)	**^{14}C Age** (yr)	**±1σ** (s.d.)	**Reservoir Age** (yr)	**±1σ** (s.d.)	**CALIB Age** (yr [MARINE13])	**±1σ** (s.d.)	**Δ^{14}C** (‰)	**Sed. Rate** (cm/kyr)
2--3	*n.d.*	*n.d.*	*n.d.*	*n.d.*	*n.d.*	*n.d.*	*n.d.*	*n.d.*
12--13	1,271	70	500	40	668	73	-146	15.0
24--25	1,926	74	540	50	1275	89	-213	19.8
50--51	3,696	82	500	45	3421	113	-369	12.1
65--66	8,048	103	490	50	8355	124	-633	3.0
80--81a	*n.d.*	*n.d.*	*n.d.*	*n.d.*	*n.d.*	*n.d.*	*n.d.*	*n.d.*
80--81b	10,296	106	485	60	11140	154	-722	5.4
84--85	10,445	96	485	65	11367	196	-728	17.6
88--89	10,962	94	485	65	12273	192	-745	4.4
95--96	11,180	91	485	70	12549	122	-751	25.4
111--112	11,915	118	545	100	13172	160	-773	25.7
119--120	12,538	128	575	80	13761	178	-790	11.9
141--142	13,449	111	695	125	15021	335	-813	18.3
150--151	13,588	113	695	125	15295	284	-816	32.8
154--155a	*n.d.*	*n.d.*	*n.d.*	*n.d.*	*n.d.*	*n.d.*	*n.d.*	*n.d.*
154--155b	13,924	117	735	105	15724	252	-823	9.3
158--159	14,076	137	735	105	15942	248	-827	18.3
170--171	14,540	143	770	85	16553	259	-836	19.6
174--175	14,649	154	790	85	16681	275	-839	*n.d.*
178--179	14,715	123	800	90	16759	246	-840	64.1
193--194	*n.d.*	*n.d.*	*n.d.*	*n.d.*	*n.d.*	*n.d.*	*n.d.*	*n.d.*
280--281	19,673	185	1655	125	21728	307	-914	20.5

SHAK06-5K	*Bulk TOC*					
**Core Depth** (cm)	**^{14}C Age** (yr)	**±1σ** (s.d.)	**Δ^{14}C** (‰)	***d_{50}*** (µm)	**TOC$_{\%}$**	**C/N**
2--3	2,156	50	-235	5.7	0.57	5.5
12--13	2,634	51	-280	5.5	*n.d.*	5.9
24--25	3,166	52	-326	5.8	0.71	*n.d.*
50--51	5,033	56	-466	5.4	0.72	*n.d.*
65--66	8,619	69	-658	5.1	0.78	*n.d.*
80--81a	11,603	57	-764	6.1	0.67	6.3
80--81b	11,626	63	-765	*n.d.*	*n.d.*	*n.d.*
84--85	11,861	58	-772	6.4	0.66	6.5
88--89	12,148	58	-780	6.9	0.63	6.5
95--96	12,695	45	-794	6.1	0.70	5.9
111--112	13,651	52	-817	4.6	0.68	6.0
119--120	13,996	50	-825	*n.d.*	0.62	5.5
141--142	14,773	70	-841	6.0	0.61	*n.d.*
150--151	15,103	53	-847	6.3	0.59	6.0
154--155a	15,819	57	-860	*n.d.*	0.57	5.9
154--155b	15,851	71	-861	*n.d.*	*n.d.*	*n.d.*
158--159	15,724	130	-859	6.3	0.54	*n.d.*
170--171	16,143	71	-866	6.0	0.58	6.5
174--175	16,140	66	-866	6.1	0.64	6.7
178--179	17,026	78	-880	5.9	0.69	6.7
193--194	16,142	79	-866	5.7	0.68	*n.d.*
280--281	20,923	104	-926	*n.d.*	0.62	6.5

SHAK06-5K	*Clay TOC*					
**Core Depth** (cm)	**^{14}C Age** (yr)	**±1σ** (s.d.)	**Δ^{14}C** (‰)	**TOC$_{\%}$**	**Bulk$_{\%}$** (relative)	**Bulk$_{TOC\%}$** (relative)
2--3	1,986	35	-219	0.56	18.7	15.6
12--13	2,429	51	-261	0.68	20.9	*n.d.*
24--25	2,808	51	-295	0.63	19.1	16.1
50--51	4,780	56	-448	0.72	20.9	20.7
65--66	8,241	66	-642	0.83	21.6	22.2
80--81a	11,298	64	-755	0.81	18.9	20.7
80--81b	11,321	63	-756	0.61	*n.d.*	*n.d.*
84--85	11,879	54	-772	0.57	18.0	15.0
88--89	12,309	57	-784	0.57	17.4	14.9
95--96	12,870	59	-799	0.50	18.9	14.4
111--112	13,861	59	-822	0.64	*n.d.*	*n.d.*
119--120	13,799	61	-821	0.49	19.0	*n.d.*
141--142	14,710	68	-840	0.46	18.6	*n.d.*
150--151	15,324	63	-852	0.43	17.8	10.0
154--155a	15,393	63	-853	0.39	*n.d.*	*n.d.*
154--155b	14,970	69	-845	0.39	*n.d.*	*n.d.*
158--159	14,309	112	-832	0.34	17.4	9.3
170--171	14,680	69	-839	0.49	17.8	13.1
174--175	15,890	65	-862	0.54	18.7	13.7
178--179	16,385	58	-870	0.46	18.9	*n.d.*
193--194	16,415	71	-870	0.42	19.6	*n.d.*
280--281	19,701	84	-914	0.49	*n.d.*	*n.d.*

SHAK06-5K	*Fine Silt TOC*					
**Core Depth** (cm)	**^{14}C Age** (yr)	**±1σ** (s.d.)	**Δ^{14}C** (‰)	**TOC$_{\%}$**	**Bulk$_{\%}$** (relative)	**Bulk$_{TOC\%}$** (relative)
2--3	2,901	35	-303	0.71	47.0	53.2
12--13	3,449	51	-349	0.77	47.0	*n.d.*
24--25	3,857	54	-381	0.71	46.4	48.0
50--51	6,117	78	-533	0.60	46.4	38.7
65--66	8,957	70	-672	0.80	46.8	46.2
80--81a	12,072	55	-778	0.76	44.6	46.1
80--81b	11,913	89	-773	0.76	*n.d.*	*n.d.*
84--85	12,572	59	-791	0.75	44.4	48.8
88--89	13,006	57	-802	0.73	42.3	46.5
95--96	13,637	49	-817	0.80	45.5	51.0
111--112	14,762	69	-841	0.80	*n.d.*	*n.d.*
119--120	14,089	87	-827	0.73	48.1	*n.d.*
141--142	15,757	76	-859	0.75	41.3	*n.d.*
150--151	17,048	75	-880	0.75	41.5	46.0
154--155a	16,832	98	-877	0.73	*n.d.*	
154--155b	*n.d.*	*n.d.*	*n.d.*	0.69	*n.d.*	*n.d.*
158--159	16,358	143	-869	0.69	45.4	48.2
170--171	16,673	84	-875	0.71	47.2	50.8
174--175	17,160	84	-882	0.80	45.5	49.4
178--179	17,337	75	-884	0.23	41.2	*n.d.*
193--194	*n.d.*	*n.d.*	*n.d.*	0.79	45.4	*n.d.*
280--281	20,745	93	-924	0.81	*n.d.*	*n.d.*

SHAK06-5K	*Coarse Silt TOC*						
**Core Depth** (cm)	**^{14}C Age** (yr)	**±1σ** (s.d.)	**Δ^{14}C** (‰)	**Avg. GS** (µm)	**TOC$_{\%}$**	**Bulk$_{\%}$** (relative)	**Bulk$_{TOC\%}$** (relative)
2--3	3,033	46	-314	24.1	0.52	30.0	26.6
12--13	4,247	56	-411	23.6	0.57	28.8	*n.d.*
24--25	4,581	60	-435	25.3	0.61	30.5	26.5
50--51	*n.d.*	*n.d.*	*n.d.*	24.4	0.82	29.5	33.3
65--66	9,157	83	-680	24.9	0.69	27.8	23.6
80--81a	12,169	62	-780	25.3	0.62	33.1	27.9
80--81b	*n.d.*	*n.d.*	*n.d.*	*n.d.*	*n.d.*	*n.d.*	*n.d.*
84--85	12,485	61	-789	25.3	0.63	33.7	30.8
88--89	13,311	68	-809	25.6	0.60	35.9	32.2
95--96	13,615	63	-816	24.4	0.63	32.2	28.9
111--112	14,195	52	-829	*n.d.*	0.64	*n.d.*	*n.d.*
119--120	14,251	62	-830	23.7	*n.d.*	25.4	*n.d.*
141--142	*n.d.*	*n.d.*	*n.d.*	25.8	*n.d.*	36.5	*n.d.*
150--151	16,124	86	-866	25.0	0.66	37.5	38.4
154--155a	15,770	70	-860	*n.d.*	0.65	*n.d.*	*n.d.*
154--155b	*n.d.*	*n.d.*	*n.d.*	*n.d.*	*n.d.*	*n.d.*	*n.d.*
158--159	16,789	150	-876	24.8	0.65	33.6	33.9
170--171	17,188	74	-882	24.1	0.60	31.6	28.8
174--175	17,077	70	-881	24.7	0.66	31.9	28.5
178--179	17,549	81	-887	24.2	*n.d.*	36.8	*n.d.*
193--194	*n.d.*	*n.d.*	*n.d.*	24.7	*n.d.*	31.2	*n.d.*
280--281	21,409	84	-930	*n.d.*	*n.d.*	*n.d.*	*n.d.*

SHAK06-5K	*Sand TOC*					
**Core Depth** (cm)	**^{14}C Age** (yr)	**±1σ** (s.d.)	**Δ^{14}C** (‰)	**TOC$_{\%}$**	**Bulk$_{\%}$** (relative)	**Bulk$_{TOC\%}$** (relative)
2--3	2,089	45	-229	0.86	4.4	4.61
12--13	1,378	35	-158	1.35	3.3	*n.d.*
24--25	3,293	53	-336	1.46	4.1	9.35
50--51	5,584	63	-501	1.65	3.2	7.32
65--66	8,789	75	-665	1.73	3.7	7.99
80--81a	9,320	44	-687	1.13	3.4	5.27
80--81b	*n.d.*	*n.d.*	*n.d.*	*n.d.*	*n.d.*	*n.d.*
84--85	10,698	56	-736	0.96	3.9	5.41
88--89	11,412	46	-758	0.98	4.4	6.43
95--96	11,721	49	-768	1.21	3.3	5.68
111--112	13,013	48	-802	1.14	*n.d.*	*n.d.*
119--120	12,812	100	-797	1.43	3.4	*n.d.*
141--142	14,849	134	-843	1.30	3.6	*n.d.*
150--151	14,577	64	-837	1.18	3.2	5.61
154--155a	15,214	53	-850	1.07	*n.d.*	*n.d.*
154--155b	16,678	153	-875	1.19	*n.d.*	*n.d.*
158--159	15,965	149	-863	1.58	3.6	8.68
170--171	14,306	59	-832	1.42	3.4	7.32
174--175	13,996	52	-825	1.56	4.0	8.42
178--179	*n.d.*	*n.d.*	*n.d.*	*1.51*	3.1	*n.d.*
193--194	14,576	123	-837	1.02	3.7	*n.d.*
280--281	19,620	207	-913	*n.d.*	*n.d.*	*n.d.*

Supplementary Table 2: X-ray fluorescence (XRF) analyses were measured for (sub)cores of SHAK06-5K on a Avaatech XRF scanner (University of Cambridge) at 0.5 cm depth intervals. Cores were analyzed with 0.2 mA current at three different voltages: 10 kV, 30 kV (lead filter), and 50 kV (copper filter).

Core Depth		Aluminium	Calcium	Titanium	Zirconium
(cm)		(area)	(area)	(area)	(area)
0.5		38673	5.16E+05	28776	1162
1		40640	6.11E+05	33896	1316
1.5		38765	5.29E+05	30568	1190
2		39295	5.56E+05	31725	1163
2.5		40692	5.79E+05	33214	1208
3		41120	6.18E+05	33666	1188
3.5		41020	6.19E+05	33167	1196
4		42050	6.44E+05	33119	1113
4.5		42944	6.77E+05	32897	1193
5		43055	6.73E+05	33479	1252
5.5		43499	6.91E+05	33794	1214
6		42879	6.84E+05	33477	1170
6.5		41588	7.05E+05	33626	1248
7		43713	7.35E+05	34155	1221
7.5		45911	7.82E+05	34899	1292
8		47690	8.34E+05	35006	1353
8.5		49970	8.72E+05	35935	1304
9		50330	8.65E+05	36126	1191
9.5		50771	8.25E+05	37025	1264
10		50263	8.22E+05	35872	1244
10.5		49878	8.25E+05	36617	1256
11		49445	8.38E+05	35669	1199
11.5		49170	8.65E+05	36256	1162
12		50234	8.80E+05	36172	1252
12.5		50458	8.83E+05	35311	1332
12.5		50435	8.86E+05	36256	1288
12.5		49954	8.87E+05	35632	1257
13		49867	8.69E+05	35902	1342
13.5		51219	8.78E+05	35610	1222
14		50742	8.91E+05	35770	1303
14.5		50483	8.83E+05	35644	1266
15		49825	8.72E+05	36296	1268
15.5		49908	8.79E+05	35590	1241
16		50097	8.96E+05	36474	1350
16.5		50881	9.13E+05	35706	1303
17		50319	9.17E+05	37041	1214
17.5		49804	9.02E+05	36874	1416
18		49510	9.39E+05	37805	1258
18.5		48862	9.34E+05	37235	1310
19		47548	9.13E+05	37642	1268
19.5		49054	9.38E+05	37929	1313
20		49710	9.37E+05	38040	1301

Core Depth		Aluminium	Calcium	Titanium	Zirconium
20.5		48826	9.50E+05	37159	1352
21		47928	9.38E+05	36559	1267
21.5		46879	9.65E+05	34079	1116
22		39718	1.16E+06	25664	1061
22.5		40189	1.08E+06	26783	1136
23		45173	9.94E+05	32073	1213
23.5		45495	9.45E+05	34017	1192
24		46217	9.60E+05	33317	1297
24.5		47670	9.70E+05	34138	1232
25		48176	9.72E+05	34179	1263
25		48316	9.77E+05	34859	1306
25		47507	9.79E+05	34616	1289
25.5		46547	9.45E+05	34289	1291
26		46663	9.51E+05	33348	1305
26.5		47927	9.70E+05	33972	1347
27		48249	9.59E+05	32516	1236
27.5		47860	9.64E+05	32080	1310
28		47630	9.69E+05	32332	1347
28.5		48378	9.45E+05	32445	1323
29		47967	9.33E+05	33328	1171
29.5		47788	9.30E+05	32461	1302
30		47727	9.31E+05	33236	1256
30.5		48654	9.40E+05	33236	1207
31		48436	9.18E+05	32840	1228
31.5		47883	8.90E+05	33031	1298
32		47050	9.15E+05	31922	1272
32.5		48169	9.63E+05	32482	1301
33		48187	9.48E+05	32811	1292
33.5		48054	9.33E+05	31815	1247
34		48248	9.15E+05	32180	1165
34.5		48308	8.90E+05	32092	1212
35		47795	8.90E+05	32333	1220
35.5		48264	9.26E+05	31936	1281
36		48905	9.56E+05	31795	1175
36.5		48570	9.52E+05	32376	1190
37		48414	9.47E+05	30855	1343
37.5		48306	9.42E+05	31751	1210
37.5		47321	9.40E+05	31254	1359
37.5		46959	9.35E+05	31126	1250
38		47397	9.39E+05	32162	1269
38.5		48315	9.50E+05	31722	1199
39		47637	9.52E+05	31630	1148
39.5		47991	9.76E+05	30551	1166
40		48368	9.81E+05	30826	1287

Core Depth		Aluminium	Calcium	Titanium	Zirconium
40.5		48379	9.49E+05	31177	1320
41		47551	9.18E+05	31523	1199
41.5		48331	9.32E+05	31768	1171
42		49118	9.40E+05	31762	1123
42.5		48891	9.46E+05	31618	1268
43		48576	9.52E+05	31378	1198
43.5		48041	9.23E+05	31360	1182
44		48771	9.31E+05	32956	1242
44.5		48839	9.35E+05	33068	1304
45		48279	9.16E+05	32351	1230
45.5		47195	9.05E+05	31486	1245
46		46744	9.20E+05	31884	1238
46.5		47411	9.30E+05	31392	1216
47		48704	9.10E+05	32273	1268
47.5		48498	9.37E+05	32062	1258
48		47815	9.56E+05	31832	1271
48.5		48127	9.51E+05	31530	1249
49		47466	9.48E+05	32107	1219
49.5		47532	9.48E+05	30758	1210
50		47651	9.48E+05	31619	1187
50		46850	9.44E+05	32190	1237
50		45632	9.38E+05	31992	1207
50.5		45329	8.75E+05	32836	1303
51		46349	8.91E+05	31851	1233
51.5		46256	9.26E+05	30524	1262
52		44972	8.65E+05	31272	1236
52.5		45137	8.77E+05	31700	1161
53		46435	9.35E+05	31085	1211
53.5		47293	9.52E+05	31842	1160
54		46800	9.53E+05	31245	1242
54.5		47210	9.46E+05	32308	1199
55		47603	9.27E+05	31705	1183
55.5		46435	9.37E+05	31823	1232
56		46796	9.44E+05	31400	1194
56.5		46453	9.62E+05	30784	1172
57		47203	9.78E+05	30632	1162
57.5		46684	1.02E+06	30189	1181
58		45004	1.05E+06	29055	1053
58.5		45041	1.07E+06	28794	1122
59		45464	1.10E+06	29201	1127
59.5		46584	1.11E+06	29162	1085
60		45140	1.14E+06	28565	1135

Core Depth		Aluminium	Calcium	Titanium	Zirconium
60.5		45139	1.13E+06	29003	1164
61		44993	1.09E+06	29278	1172
61.5		45705	1.09E+06	30030	1227
62		45550	1.12E+06	29310	1176
62.5		46402	1.08E+06	29444	1152
62.5		45334	1.08E+06	29717	1148
62.5		44974	1.08E+06	29582	1202
63		45270	1.03E+06	31211	1203
63.5		46008	9.84E+05	30821	1149
64		44999	9.98E+05	30362	1178
64.5		45620	1.02E+06	30122	1053
65		44992	1.10E+06	29114	1126
65.5		44247	1.14E+06	28894	1054
66		44053	1.12E+06	29060	1200
66.5		44689	1.06E+06	30448	1091
67		45841	1.02E+06	30436	1062
67.5		43468	9.95E+05	30110	1074
68		42938	1.01E+06	30154	1186
68.5		44142	1.03E+06	29880	1141
69		43744	1.05E+06	29823	1192
69.5		44287	1.03E+06	31550	1295
70		45209	1.00E+06	31562	1185
70.5		44965	1.00E+06	32508	1220
71		44473	1.03E+06	30814	1199
71.5		45128	1.04E+06	30652	1094
72		44606	1.03E+06	30421	1157
72.5		44647	1.05E+06	29801	1187
73		44877	1.02E+06	30868	1151
73.5		45335	1.02E+06	31240	1178
74		45183	1.01E+06	31131	1250
74.5		46102	9.91E+05	32827	1236
75		46136	9.99E+05	32365	1216
75		45497	1.00E+06	33184	1237
75		44373	9.99E+05	32975	1226
75.5		44961	9.67E+05	33740	1239
76		46518	1.02E+06	32815	1247
76.5		47123	9.89E+05	33487	1202
77		48336	9.72E+05	34883	1210
77.5		47568	9.81E+05	34935	1249
78		46928	9.76E+05	35019	1390
78.5		47319	9.27E+05	35683	1379
79		46062	9.39E+05	34911	1335
79.5		46235	9.23E+05	34360	1357
80		46876	9.28E+05	35116	1362

Core Depth		Aluminium	Calcium	Titanium	Zirconium
80.5		45914	9.30E+05	32790	1306
81		46677	9.78E+05	33028	1426
81.5		46036	9.70E+05	33839	1386
82		47094	8.71E+05	36290	1327
82.5		47669	8.15E+05	36701	1491
83		45219	7.16E+05	37271	1581
83.5		46349	7.97E+05	37633	1407
84		48591	8.18E+05	37943	1483
84.5		47177	7.48E+05	38865	1525
85		47522	7.73E+05	38766	1561
85.5		47063	7.87E+05	38303	1484
86		47104	8.34E+05	38790	1384
86.5		48174	8.11E+05	40018	1519
87		49730	8.24E+05	39747	1490
87.5		47836	7.89E+05	38184	1487
87.5		46546	7.86E+05	38849	1581
87.5		45750	7.79E+05	38505	1607
88		44817	7.76E+05	37039	1529
88.5		45102	7.67E+05	37026	1494
89		44448	7.62E+05	36432	1535
89.5		41805	7.25E+05	35684	1511
90		40887	7.50E+05	35475	1583
90.5		46663	8.45E+05	38374	1575
91		48273	8.83E+05	38120	1491
91.5		48170	9.00E+05	38261	1490
92		48919	9.21E+05	38026	1529
92.5		47589	8.84E+05	37614	1556
93		44454	8.50E+05	36392	1383
93.5		47301	8.61E+05	38006	1412
94		46826	9.28E+05	37652	1328
94.5		48882	9.62E+05	36909	1431
95		48714	9.25E+05	36521	1509
95.5		48639	8.96E+05	36711	1449
96		48449	9.22E+05	36400	1393
96.5		48563	9.51E+05	35829	1280
97		48200	9.66E+05	35145	1329
97.5		47756	9.47E+05	36295	1393
98		48960	9.43E+05	36999	1447
98.5		48959	9.17E+05	36774	1519
99		46331	8.94E+05	35366	1431
99.5		46272	8.75E+05	36336	1649
100		49570	9.15E+05	37116	1449

Core Depth		Aluminium	Calcium	Titanium	Zirconium
100		49557	9.19E+05	37531	1380
100		49179	9.19E+05	37657	1391
100.5		47544	9.32E+05	35658	1357
101		46540	8.82E+05	35742	1292
101.5		48038	9.13E+05	36494	1394
102		49142	8.87E+05	36881	1379
102.5		50021	8.80E+05	37175	1392
103		47743	8.81E+05	36302	1462
103.5		47526	8.47E+05	36473	1261
104		48115	8.52E+05	37380	1227
104.5		48326	8.76E+05	37603	1293
105		49214	9.02E+05	37003	1360
105.5		49452	9.01E+05	37789	1369
106		48913	8.90E+05	37180	1247
106.5		47976	9.08E+05	36823	1321
107		47505	9.10E+05	36122	1411
107.5		48604	8.80E+05	37167	1294
108		50331	8.93E+05	37728	1332
108.5		49640	8.69E+05	37502	1316
109		48965	8.72E+05	37309	1313
109.5		48595	8.65E+05	37073	1399
110		48454	8.45E+05	36433	1285
110.5		47931	8.69E+05	36086	1318
111		48322	9.17E+05	35801	1312
111.5		48526	8.89E+05	36757	1390
112		48970	8.65E+05	37311	1323
112.5		48686	8.32E+05	37862	1397
112.5		47935	8.35E+05	37788	1372
112.5		46733	8.34E+05	37058	1448
113		47746	8.15E+05	37910	1356
113.5		48545	8.20E+05	37077	1259
114		50001	7.90E+05	37624	1296
114.5		48990	7.95E+05	37326	1422
115		48394	7.73E+05	37534	1291
115.5		48480	7.49E+05	37595	1231
116		48208	7.61E+05	37534	1299
116.5		47889	7.46E+05	37995	1428
117		48385	7.87E+05	37338	1293
117.5		48142	8.06E+05	37343	1353
118		47868	7.47E+05	37792	1293
118.5		49822	7.49E+05	39758	1407
119		50232	7.59E+05	39496	1383
119.5		50544	7.60E+05	40018	1246
120		50354	7.41E+05	40098	1448

Core Depth		Aluminium	Calcium	Titanium	Zirconium
120.5		49497	7.49E+05	39569	1359
121		49055	7.36E+05	39418	1424
121.5		48107	8.16E+05	38077	1450
122		47940	8.40E+05	38027	1328
122.5		48702	7.63E+05	39537	1414
123		49546	7.26E+05	38966	1331
123.5		48751	7.43E+05	38746	1360
124		49255	7.00E+05	39337	1297
124.5		49088	6.94E+05	38813	1387
125		48843	7.08E+05	39050	1401
125		48541	7.11E+05	38833	1391
125		47531	7.12E+05	39709	1393
125.5		48475	7.00E+05	39590	1391
126		49827	7.07E+05	39430	1502
126.5		49967	6.98E+05	39447	1562
127		49370	7.19E+05	39010	1399
127.5		49615	7.25E+05	38921	1441
128		49798	7.14E+05	39713	1487
128.5		50170	6.94E+05	39118	1509
129		48611	6.94E+05	38366	1518
129.5		48867	6.97E+05	37883	1564
130		48809	7.34E+05	37156	1327
130.5		49884	7.40E+05	37613	1373
131		48459	7.09E+05	38321	1513
131.5		48680	6.95E+05	39676	1468
132		48206	6.93E+05	38130	1573
132.5		49703	6.71E+05	38211	1602
133		48741	6.84E+05	38201	1492
133.5		48019	6.67E+05	38436	1507
134		47971	6.55E+05	37767	1529
134.5		46640	6.39E+05	37206	1632
135		47977	6.94E+05	35876	1499
135.5		48936	6.69E+05	37349	1497
136		48743	6.38E+05	37464	1544
136.5		49328	6.77E+05	37465	1465
137		49168	6.59E+05	37808	1508
137.5		49369	6.68E+05	39072	1619
137.5		49170	6.73E+05	38177	1465
137.5		49225	6.74E+05	38324	1642
138		48049	6.68E+05	38446	1593
138.5		49384	6.69E+05	38779	1648
139		50055	6.74E+05	38450	1627
139.5		48346	6.71E+05	39552	1649
140		48573	6.73E+05	38933	1632

Core Depth		Aluminium	Calcium	Titanium	Zirconium
140.5		47352	6.73E+05	38163	1600
141		48479	6.91E+05	39389	1639
141.5		47608	6.96E+05	39026	1587
142		45237	6.85E+05	37110	1535
142.5		44695	6.99E+05	37016	1549
143		45642	7.12E+05	38570	1551
143.5		45860	7.10E+05	38731	1660
144		45087	7.06E+05	38672	1558
144.5		44299	7.36E+05	36240	1556
145		42784	7.27E+05	36249	1503
145.5		42257	7.37E+05	34520	1649
146		43209	7.68E+05	33656	1497
146.5		44319	7.97E+05	33641	1489
147		42869	8.26E+05	31968	1655
147.5		41985	9.36E+05	30296	1380
148		44799	7.77E+05	34766	1555
148.5		46649	7.05E+05	37744	1460
149		45001	6.57E+05	37287	1606
149.5		42603	7.41E+05	33963	1527
150		46770	7.69E+05	38707	1762
150		45533	7.64E+05	38372	1731
150		45190	7.59E+05	37949	1619
150.5		44906	7.26E+05	39200	1673
151		36455	3.98E+05	26839	1235
151.5		40946	5.97E+05	33640	1462
152		42637	6.07E+05	35082	1480
152.5		41285	5.54E+05	33675	1498
153		43036	5.60E+05	34212	1473
153.5		44376	5.58E+05	35548	1371
154		44811	5.39E+05	33526	1493
154.5		44552	5.42E+05	34352	1425
155		44736	5.49E+05	33840	1381
155.5		44673	5.68E+05	34034	1505
156		46480	6.03E+05	35255	1461
156.5		45330	5.55E+05	35093	1443
157		44095	5.69E+05	35080	1475
157.5		45179	5.66E+05	34846	1425
158		45162	5.62E+05	34838	1441
158.5		43519	5.85E+05	34326	1522
159		45561	5.91E+05	35678	1557
159.5		47546	5.73E+05	36831	1583
160		49518	5.92E+05	37440	1597

Core Depth		Aluminium	Calcium	Titanium	Zirconium
160.5		49030	5.74E+05	38326	1624
161		49450	5.71E+05	38115	1464
161.5		49618	5.43E+05	37520	1425
162		49188	5.60E+05	37285	1551
162.5		50141	5.84E+05	37954	1659
163		49968	5.51E+05	37619	1518
163.5		49413	5.49E+05	37592	1573
163.5		48398	5.48E+05	37376	1525
163.5		48414	5.46E+05	37540	1602
164		48077	5.53E+05	38560	1513
164.5		49521	5.69E+05	38485	1527
165		49885	5.86E+05	38253	1651
165.5		49824	5.78E+05	37468	1449
166		49379	5.30E+05	37880	1482
166.5		47548	4.68E+05	36560	1491
167		47671	4.77E+05	37114	1461
167.5		49836	5.23E+05	39591	1480
168		50007	5.62E+05	38950	1431
168.5		50441	5.93E+05	38719	1480
169		50574	6.07E+05	39234	1427
169.5		49454	6.08E+05	37650	1463
170		49924	6.28E+05	37625	1458
170.5		47774	6.47E+05	37060	1464
171		47406	6.77E+05	36755	1364
171.5		46531	7.39E+05	36240	1360
172		47225	7.59E+05	35371	1441
172.5		46578	7.42E+05	36668	1359
173		47653	7.06E+05	37879	1568
173.5		48869	6.43E+05	39806	1563
174		46882	6.61E+05	39310	1700
174.5		43411	7.38E+05	36348	1407
175		39904	6.09E+05	36005	1605
175.5		37517	5.40E+05	35167	1509
176		39161	6.54E+05	35843	1460
176		39912	6.71E+05	37160	1426
176		40457	6.75E+05	36613	1455
176.5		41805	7.16E+05	36848	1452
177		42494	7.57E+05	36699	1577
177.5		44104	7.87E+05	36529	1458
178		45680	8.03E+05	36924	1463
178.5		45987	7.82E+05	37305	1399
179		44791	8.15E+05	36753	1578
179.5		46710	8.38E+05	36150	1467
180		47131	8.25E+05	36306	1375

Core Depth		Aluminium	Calcium	Titanium	Zirconium
180.5		46369	8.41E+05	36914	1393
181		46505	8.50E+05	36756	1426
181.5		46765	8.35E+05	35413	1387
182		47267	7.76E+05	37096	1386
182.5		45815	7.87E+05	36265	1354
183		45360	8.03E+05	36212	1307
183.5		46059	8.13E+05	35518	1398
184		46229	8.39E+05	35363	1314
184.5		46364	8.10E+05	35564	1282
185		45919	7.94E+05	34556	1277
185.5		46194	8.09E+05	33813	1359
186		46635	8.18E+05	34978	1391
186.5		46565	8.22E+05	34587	1275
187		46540	8.30E+05	33819	1280
187.5		46324	8.38E+05	34039	1249
188		44877	8.22E+05	34037	1343
188.5		45346	8.30E+05	34005	1335
188.5		45881	8.34E+05	33777	1285
188.5		44916	8.35E+05	34109	1361
189		44211	8.07E+05	33981	1367
189.5		43424	8.13E+05	32864	1295
190		46133	8.29E+05	33634	1335
190.5		45931	8.33E+05	32934	1277
191		46424	8.51E+05	33605	1452
191.5		46119	8.54E+05	32151	1304
192		45910	8.40E+05	33277	1338
192.5		45553	8.41E+05	32961	1338
193		45983	8.43E+05	33656	1355
193.5		45786	8.37E+05	33600	1338
194		46048	8.48E+05	33223	1273
194.5		45229	8.51E+05	33487	1267
195		45579	8.15E+05	33163	1371
195.5		45660	8.38E+05	34630	1362
196		46475	8.40E+05	34029	1352
196.5		45959	8.52E+05	34097	1344
197		45802	8.65E+05	33470	1430
197.5		46817	8.58E+05	34567	1413
198		46886	8.33E+05	34130	1371
198.5		46606	8.40E+05	34810	1355
199		46869	8.57E+05	34594	1317
199.5		47130	8.71E+05	34525	1347
200		45857	8.51E+05	34967	1332

Core Depth		Aluminium	Calcium	Titanium	Zirconium
200.5		44142	8.47E+05	35336	1377
201		44894	8.25E+05	34876	1428
201		43731	8.18E+05	34467	1324
201		43109	8.11E+05	34121	1490
201.5		45732	8.49E+05	34902	1478
202		47428	8.77E+05	35631	1385
202.5		46038	8.98E+05	36485	1448
203		43719	8.69E+05	35789	1430
203.5		42762	8.39E+05	34728	1337
204		42396	8.28E+05	34133	1305
204.5		42160	8.33E+05	34158	1286
205		43895	8.85E+05	36442	1416
205.5		46388	8.96E+05	36189	1322
206		46217	8.93E+05	34955	1429
206.5		46862	8.95E+05	35058	1366
207		47397	8.96E+05	34578	1347
207.5		46529	9.17E+05	35162	1367
208		47173	9.07E+05	34246	1260
208.5		46164	9.22E+05	33314	1311
209		46049	8.89E+05	33736	1361
209.5		47165	9.08E+05	33293	1372
210		46009	9.17E+05	33402	1216
210.5		45656	9.03E+05	32769	1327
211		45792	8.60E+05	32475	1360
211.5		46210	8.65E+05	33458	1271
212		46497	8.72E+05	33257	1265
212.5		46335	8.78E+05	32252	1327
213		46631	8.66E+05	33046	1254
213.5		46667	8.64E+05	33344	1318
213.5		46078	8.67E+05	32876	1327
213.5		45681	8.67E+05	33482	1226
214		45303	8.77E+05	32384	1251
214.5		45471	8.91E+05	32033	1333
215		45333	8.68E+05	31636	1257
215.5		45740	8.07E+05	32816	1332
216		45339	7.80E+05	32540	1273
216.5		44786	7.90E+05	32301	1326
217		44527	7.88E+05	31871	1332
217.5		44901	8.38E+05	31525	1302
218		45282	8.48E+05	32016	1354
218.5		44693	8.32E+05	32831	1285
219		45119	8.38E+05	32746	1321
219.5		44795	8.16E+05	32396	1267
220		45231	8.19E+05	32957	1242

Core Depth		Aluminium	Calcium	Titanium	Zirconium
220.5		44511	7.95E+05	32675	1285
221		45248	8.41E+05	33125	1222
221.5		45097	8.41E+05	33240	1330
222		45449	8.47E+05	32618	1227
222.5		45367	8.68E+05	32568	1293
223		45482	8.37E+05	32813	1325
223.5		45263	8.06E+05	33444	1341
224		45008	8.46E+05	33718	1196
224.5		45981	8.45E+05	33542	1277
225		45659	8.44E+05	32170	1317
225.5		44830	8.58E+05	31868	1277
226		45742	8.52E+05	31080	1379
226		45276	8.59E+05	32157	1309
226		45237	8.58E+05	32115	1364
226.5		44708	8.17E+05	32031	1258
227		45373	8.22E+05	31814	1277
227.5		44549	8.38E+05	31068	1283
228		46575	8.40E+05	32908	1258
228.5		46973	8.33E+05	32119	1292
229		46828	7.87E+05	34087	1272
229.5		46292	8.24E+05	32847	1334
230		46680	8.28E+05	34291	1294
230.5		46503	8.16E+05	34198	1298
231		46881	8.42E+05	34552	1208
231.5		45010	8.43E+05	34026	1339
232		43171	8.24E+05	33447	1296
232.5		44609	8.40E+05	33848	1360
233		44348	8.29E+05	33644	1381
233.5		43118	8.41E+05	32698	1395
234		44914	8.49E+05	33333	1315
234.5		44051	8.44E+05	32932	1324
235		44623	8.51E+05	33447	1355
235.5		44386	8.34E+05	32630	1355
236		43507	8.16E+05	33701	1316
236.5		42175	8.13E+05	31906	1345
237		42255	8.14E+05	31932	1301
237.5		44996	8.06E+05	31850	1167
238		43481	7.47E+05	30184	1117
238.5		43870	7.68E+05	31526	1215
238.5		42782	7.65E+05	30695	1329
238.5		42596	7.60E+05	30776	1287
239		41700	7.39E+05	30581	1317
239.5		40420	7.16E+05	29720	1341
240		41608	7.37E+05	30691	1281

Core Depth		Aluminium	Calcium	Titanium	Zirconium
240.5		45130	7.81E+05	31493	1237
241		47354	7.87E+05	32510	1251
241.5		45960	7.93E+05	30954	1233
242		45370	8.11E+05	30093	1345
242.5		45580	8.21E+05	31460	1293
243		46817	8.37E+05	30995	1320
243.5		46996	7.98E+05	33552	1382
244		46935	8.31E+05	32548	1317
244.5		47246	8.08E+05	33611	1433
245		47422	8.12E+05	33164	1288
245.5		47068	8.29E+05	32140	1318
246		46809	8.46E+05	31554	1309
246.5		46841	8.39E+05	32369	1245
247		47599	8.04E+05	33519	1451
247.5		47770	7.86E+05	33101	1285
248		47815	8.11E+05	33530	1406
248.5		47475	7.49E+05	32961	1399
249		46162	7.53E+05	33673	1337
249.5		45315	7.51E+05	33632	1322
250		46040	7.74E+05	33382	1320
250.5		44702	7.73E+05	32587	1233
251		42997	7.67E+05	31047	1302
251		42339	7.54E+05	30815	1258
251		41417	7.38E+05	30396	1268
251.5		37289	7.08E+05	29205	1313
252		37945	7.54E+05	31009	1345
252.5		42836	7.70E+05	31611	1246
253		45710	7.78E+05	32719	1283
253.5		45014	7.81E+05	31811	1345
254		43968	7.85E+05	32003	1330
254.5		43723	7.52E+05	31943	1289
255		43971	7.77E+05	32994	1257
255.5		44102	7.72E+05	33324	1307
256		43720	7.38E+05	33066	1304
256.5		44657	7.53E+05	32890	1355
257		44410	7.71E+05	32812	1351
257.5		43375	7.76E+05	33015	1329
258		45525	7.70E+05	34284	1257
258.5		44908	7.88E+05	32767	1292
259		44133	7.77E+05	32773	1312
259.5		45483	7.81E+05	33951	1281
260		45693	7.94E+05	33592	1315

Core Depth		Aluminium	Calcium	Titanium	Zirconium
260.5		45424	7.84E+05	33589	1302
261		46231	7.89E+05	33304	1351
261.5		45157	7.98E+05	33077	1357
262		45737	8.01E+05	33741	1237
262.5		45644	8.05E+05	32332	1264
263		46350	8.03E+05	31928	1317
263.5		46334	8.05E+05	33150	1253
263.5		46114	8.09E+05	32881	1318
263.5		44549	8.04E+05	32965	1253
264		45037	7.95E+05	33390	1271
264.5		45686	8.00E+05	32456	1356
265		45669	8.10E+05	32635	1268
265.5		46141	7.92E+05	32891	1356
266		46260	7.99E+05	33556	1224
266.5		45320	8.14E+05	33462	1254
267		44654	8.17E+05	33172	1284
267.5		43810	8.34E+05	33239	1285
268		40852	7.55E+05	31417	1278
268.5		42821	8.11E+05	33237	1340
269		43473	8.27E+05	33547	1349
269.5		42512	8.06E+05	33651	1237
270		41922	8.03E+05	33548	1346
270.5		43829	8.16E+05	32845	1361
271		44978	8.17E+05	32898	1301
271.5		44739	8.19E+05	33535	1218
272		44849	8.13E+05	33211	1310
272.5		45369	8.20E+05	34267	1316
273		44754	8.31E+05	33232	1282
273.5		45052	8.37E+05	33551	1308
274		45383	8.26E+05	33692	1290
274.5		45723	8.06E+05	32979	1310
275		45760	8.23E+05	33750	1363
275.5		45740	8.24E+05	33764	1234
276		46132	8.20E+05	34150	1362
276		45788	8.26E+05	34038	1330
276		45735	8.26E+05	34357	1268
276.5		44861	8.02E+05	33518	1289
277		44716	7.95E+05	33846	1350
277.5		46495	7.82E+05	33755	1425
278		47534	7.03E+05	35875	1405
278.5		47231	7.43E+05	34271	1329
279		46980	7.52E+05	34745	1370
279.5		47166	6.77E+05	35500	1468
280		46885	7.38E+05	35225	1498

Supplementary Table 3: The sediment chronology was determined by ^{210}Pb-dating at the University of Florida. ^{210}Pb, ^{137}Cs and ^{226}Ra activities were measured by low-background gamma detection. Unsupported ^{210}Pb activity in each sample was calculated by subtracting ^{226}Ra activity (*i.e* ., supported ^{210}Pb activity) from total ^{210}Pb activity at each level. Sediment ages were estimated by the constant initial concentration (CIC) model. We estimated the average mass sedimentation rate (MSR; mg cm^{-2} a^{-1}) as the slope of the regression of the natural log of unsupported ^{210}Pb against the cumulative mass (g cm^{-2}). Age errors were calculated using the 95% confidence interval of the MSR estimate.

**Depth**	**^{210}Pb Activity**		**^{210}Pb MDA**	**^{226}Ra Activity**		**^{226}Ra MDA**	**^{137}Cs Activity**		**^{137}Cs MDA**
(cm)	(dpm/g)	(st. dev.)	(dpm/g)	(dpm/g)	(st. dev.)	(dpm/g)	(dpm/g)	(st. dev.)	(dpm/g)
0.5	40.1	0.6	0.1	1.7	0.5	0.1	0.1	0.0	0.0
1.0	32.9	0.6	0.1	1.5	0.5	0.1	0.2	0.0	0.0
1.5	19.4	0.4	0.1	1.9	0.7	0.1	0.3	0.0	0.0
2.0	16.1	0.4	0.1	1.8	0.1	0.1	0.3	0.0	0.0
2.5	15.7	0.4	0.1	1.9	0.2	0.1	0.2	0.0	0.0
3.0	13.3	0.3	0.1	1.8	0.1	0.1	0.2	0.0	0.0
3.5	12.7	0.2	0.1	1.9	0.7	0.1	0.2	0.0	0.0
4.0	9.9	0.2	0.1	1.9	0.2	0.1	0.2	0.0	0.0
4.5	8.2	0.3	0.1	2.2	0.4	0.1	0.0	0.0	0.0
5.0	6.3	0.2	0.1	1.8	0.6	0.1	0.1	0.0	0.0
5.5	6.2	0.2	0.1	1.8	0.7	0.1	0.0	0.0	0.0
6.0	3.8	0.1	0.1	2.2	0.6	0.1	0.0	0.0	0.0
6.5	3.7	0.1	0.0	2.6	0.8	0.0	0.0	0.0	0.0
7.0	3.1	0.2	0.1	3.4	0.0	0.1	0.0	0.0	0.0
7.5	3.3	0.1	0.1	5.9	0.2	0.0	0.0	0.0	0.0
8.0	3.4	0.2	0.1	4.3	1.4	0.1	0.0	0.0	0.0

Depth	Excess ^{210}Pb Activity		Cumulative MSR		Sediment Age		
(cm)	(dpm/g)	(st. dev.)	(g/cm^2)	(mid-depth)	(lower)	(mid-depth)	(upper)
0.5	38.9	0.8	0.21	0.11	2010.1	2010.6	2011.1
1.0	31.7	0.8	0.45	0.33	2002.5	2004.0	2005.5
1.5	17.7	0.8	0.70	0.58	1994.2	1996.8	1999.4
2.0	14.4	0.4	0.97	0.84	1985.4	1989.1	1992.9
2.5	14.0	0.4	1.24	1.11	1976.2	1981.2	1986.1
3.0	11.6	0.4	1.53	1.39	1966.7	1972.9	1979.2
3.5	11.0	0.7	1.83	1.68	1956.8	1964.3	1971.9
4.0	8.1	0.3	2.13	1.98	1946.8	1955.7	1964.5
4.5	6.0	0.5	2.44	2.28	1936.5	1946.8	1957.0
5.0	4.6	0.6	2.76	2.60	1925.8	1937.5	1949.1
5.5	4.4	0.7	3.09	2.92	1914.8	1927.9	1941.1
6.0	1.6	0.6	3.42	3.26	1903.6	1918.2	1932.8
6.5	1.1	0.8	3.76	3.59	1892.2	1908.3	1924.4
7.0	0.0	0.2	4.11	3.94	1880.6	1898.2	1915.9
7.5	0.0	0.2	4.47	4.29	1868.7	1887.9	1907.2
8.0	0.0	1.4	4.81	4.64	1856.8	1877.6	1898.5

Supplementary Table 4: Down-core abundance (percent) of reworked calcareous nannofossils at SHAK06-5K. Nannofossil samples were prepared following particle settling protocol described in Flores and Sierro (1997). Obviously reworked fossils (i.e., Pliocene or older) were counted for at least 450 specimens per sample using polarized-light boom microscopy at 1000x magnification.

Depth(cm)	%Reworked
0	2.3
2	1.5
4	0.4
5	0.7
8	1.6
10	0.8
12	1.3
14	1.0
16	1.8
18	3.4
22	2.5
24	1.0
26	1.8
28	0.8
30	1.5
32	1.7
34	1.8
36	0.6
38	1.0
40	2.0
42	0.6
44	0.0
46	1.1
48	0.3
52	0.3
54	1.8
56	0.6
58	2.2
62	0.4
64	0.9
66	1.9
68	1.3
70	0.5
72	0.2
74	0.8
76	1.2
78	1.1
82	2.5
86	1.8
88	3.0

Depth(cm)	%Reworked
90	4.5
92	3.6
94	4.3
96	1.1
98	0.7
100	3.3
102	1.1
106	3.1
108	1.1
112	2.8
114	3.4
116	1.9
118	3.9
120	6.6
122	5.2
124	6.7
126	7.2
128	5.0
130	9.0
132	5.9
134	4.5
136	9.3
138	13.0
140	15.7
142	9.4
144	19.0
146	10.9
148	8.9
150	9.9
152	13.8
154	6.0
156	16.7
158	10.6
160	7.7
162	9.3
164	12.4
166	12.5
168	8.9
170	3.1
172	9.1
174	6.3
176	5.0
178	4.4
180	4.2
182	3.0
184	2.4
186	8.2

Depth(cm)	%Reworked
188	3.1
190	3.8
192	2.4
194	5.5
196	4.0
198	6.8
200	6.2
202	7.9
204	5.3
206	4.7
208	5.9
222	5.7
246	4.8

Supplementary references

1 Hodell, D. *et al.* A reference time scale for Site U1385 (Shackleton Site) on the SW Iberian Margin. *Global Planet. Change* **133**, 49-64 (2015).

2 Bahr, A. *et al.* Deciphering bottom current velocity and paleoclimate signals from contourite deposits in the Gulf of Cádiz during the last 140 kyr: an inorganic geochemical approach. *Geochem. Geophys. Geosy.* **15**, 3145-3160 (2014).

3 Bahr, A. *et al.* Persistent monsoonal forcing of Mediterranean Outflow Water dynamics during the late Pleistocene. *Geology* **43**, 951-954 (2015).

4 Weltje, G. J. & Tjallingii, R. Calibration of XRF core scanners for quantitative geochemical logging of sediment cores: theory and application. *Earth and Planetary Science Letters* **274**, 423-438 (2008).

5 Hodell, D. A. *et al.* The" Shackleton Site"(IODP Site U1385) on the Iberian Margin. *Sci. Drill.* **16**, 13-19 (2013).

6 Darfeuil, S. *et al.* Sea surface temperature reconstructions over the last 70 kyr off Portugal: biomarker data and regional modeling. *Paleoceanography* **31**, 40-65 (2016).

7 Salgueiro, E. *et al.* Past circulation along the western Iberian margin: a time slice vision from the Last Glacial to the Holocene. *Quaternary Sci. Rev.* **106**, 316-329 (2014).

8 Nittrouer, C., DeMaster, D., McKee, B., Cutshall, N. & Larsen, I. The effect of sediment mixing on Pb-210 accumulation rates for the Washington continental shelf. *Mar. Geol.* **54**, 201-221 (1984).
